# Gene expression analysis in RA: towards personalized medicine

**DOI:** 10.1038/tpj.2013.48

**Published:** 2014-03-04

**Authors:** A N Burska, K Roget, M Blits, L Soto Gomez, F van de Loo, L D Hazelwood, C L Verweij, A Rowe, G N Goulielmos, L G M van Baarsen, F Ponchel

**Affiliations:** 1 Leeds Institute of Rheumatic and Musculoskeletal Medicine and Leeds Musculoskeletal Biomediacal Research Unit, The University of Leeds, Leeds, UK; 2 TcLand Expression, Huningue, France; 3 Department of Pathology and Rheumatology, Inflammatory Disease Profiling Unit, VU University Medical Center, Amsterdam, The Netherlands; 4 School of law, The University of Leeds, Leeds, UK; 5 Department of Rheumatology Research and Advanced Therapeutics, Nijmegen Centre for Molecular Life Sciences, Nijmegen, The Netherlands; 6 School of Molecular and Cellular Biology, Faculty of Biological Sciences, University of Leeds, Leeds, UK; 7 Janssen Research and Development, High Wycombe, UK; 8 Molecular Medicine and Human Genetics Section, Department of Medicine, University of Crete, Heraklion, Greece; 9 Clinical Immunology and Rheumatology, Academic Medical Center, University of Amsterdam, Amsterdam, The Netherlands

**Keywords:** arthritis, gene expression, pharmacological biomarker, rheumatoid

## Abstract

Gene expression has recently been at the forefront of advance in personalized medicine, notably in the field of cancer and transplantation, providing a rational for a similar approach in rheumatoid arthritis (RA). RA is a prototypic inflammatory autoimmune disease with a poorly understood etiopathogenesis. Inflammation is the main feature of RA; however, many biological processes are involved at different stages of the disease. Gene expression signatures offer management tools to meet the current needs for personalization of RA patient's care. This review analyses currently available information with respect to RA diagnostic, prognostic and prediction of response to therapy with a view to highlight the abundance of data, whose comparison is often inconclusive due to the mixed use of material source, experimental methodologies and analysis tools, reinforcing the need for harmonization if gene expression signatures are to become a useful clinical tool in personalized medicine for RA patients.

## Introduction

Gene expression profiling is the analysis of the activity (the expression) of thousands of genes, in isolation or all at once, to create a global picture of biological functions. Many technical platforms allow the measure of the entire genome activity simultaneously in a particular cell or tissue or even organ. Gene expression profiling has recently been at the forefront of advance in personalized medicine, notably in the field of cancer and transplantation. The value of gene expression signatures was demonstrated for the prediction of tumour behaviour in several types of cancer, distinguishing groups of patients with specific tumour grades and/or prognosis,^1, 2, 3, 4, 5^ notably compared with the sole use of clinical variables in lung adenocarcinoma,^6, 7^ acute lymphoblastic leukaemia,^8^ B-cell lymphoma,^9, 10, 11^ prostate cancer,^12^ breast,^13, 14^ endometrial,^15^ colorectal,^16^ hepatocellular carcinoma^17, 18^ and other gastrointestinal cancers.^19^ Estrogen receptor status in breast tumour was reflected by specific gene expression patterns dividing high- and low-grade tumours and associated with specific biological, histological or stage features as well as metastatic propensity.^20, 21, 22^ Similar advances were also seen in organ transplantation with respect to rejection. Peripheral blood gene profiling was shown to provide adequate monitoring of immuno-suppression in individual patients who received a liver transplant^23^ as well as actually provide an alternative monitoring technology to cardiac biopsy after allograph heart transplant with significantly lower discomfort for patients.^24^ The AlloMap test for allograph heart transplant rejection has been Food and Drug Administration (FDA) approved (in 2008) in the USA, and three main clinical trials provided evidence of the usefulness of this gene expression signature.^25, 26, 27^ However, as for any other biomarkers, adoption in clinical practice is slow, and evidence that they have/will change practice are still lacking. Altogether, these results demonstrated the utility of gene expression as a modern tool able to provide personalized-specific information.

Inflammation is a biological process associated with the production of many factors, therefore suggesting that changes in gene expression could provide signatures at different phases or stages of inflammatory diseases such as rheumatoid arthritis (RA). This review will analyze currently available information with respect to RA diagnostic, prognostic and prediction of response to therapy with a view to highlight the current abundance of data, whose comparison is often inconclusive due to mixed use of a different material source, experimental methodology and statistical tools, reinforcing the need for harmonization if gene expression signature are to become a useful clinical tool in personalized medicine for RA patient's benefit.

### Gene expression as a tool to investigate pathogenesis in RA

RA remains a prototypic inflammatory autoimmune disease with a poorly understood etiopathogenesis despite recent advances in unraveling the genetic contribution to RA revealing over 50–60 risk loci.^28, 29, 30, 31^ RA is very heterogeneous as highlighted by a stronger genetic contribution to anti-citrullinated protein antibodies (ACPA)-positive disease compared with ACPA-negative disease, potentially resulting in two divergent pathogenic models^32, 33, 34^ with different rates of progression^35, 36, 37^ and response to treatment.^36, 38^

Besides these genetic advances in the understanding of RA, gene expression profiling can provide useful information to this understanding as well. Genome-wide gene expression analysis (using micro-array approaches) between RA and osteoarthritis (OA) synovial tissues have shown clear gene signatures differentiating diseases (Table 1).^39, 40^ Groups of genes associated with RA clearly identified adaptive inflammatory response-related genes, bone and cartilage degradation enzymes and transcription factors, particularly the STAT1 (signal transducer and activator of transcription 1) pathway. Several other publications also highlighted differences with diseases such as SLE (systemic lupus erythematosus), ankylosing spondylitis or psoriatic arthritis and healthy controls.^41, 42, 43^ Importantly, many genes were consistently associated with all diagnostics reflecting inflammation or autoimmune bias rather than disease specificity; others were clearly defining RA-specific events, including interleukin (IL)-7R, matrix metalloproteinase-12 (MMP-12), S100 calcium-binding protein A8, chemokines, chemokine receptors, IL-2–inducible T-cell kinase, tumour suppressor p53-binding protein, Homeo box genes, T-cell receptor, regulators of T-cell activation, CD62L Selectin and Runt-related transcription factor 3.

Heterogeneity within RA can already be associated with the progression of disease, and obvious differences have been observed in gene expression between early (<12 months) and late (>5 years) RA synovial tissue samples,^44^ suggesting involvement of different pathophysiological mechanisms over the course of disease. In a specially designed study, gene profiling was applied to identify gene signatures corresponding to different stages of RA comparing synovial biopsies from early RA (disease duration <12 months, untreated), longstanding patients RA and control synovia.^45^ Using a small microarray chip containing only 10 000 transcripts, early and longstanding RA have distinct molecular signatures with different biological processes participating at different times over the course of the disease, such as host defences, stress responses, T-cell-mediated immunity, tumour suppressor and MHC (major histocompatibility complex) class II-mediated immunity. Additional pathways involved in T-cell activation, endothelial signalling, hypoxia response and plasminogen-activating cascade were also recognized. Similar data were obtained showing differential signatures between low and high inflammatory subsets of RA patients.^46^

Gene expression profiling in established RA further revealed three main types of synovial tissue, the first with mainly T and B cells, antigen-presenting cells and MHC gene signature, a second signature included mainly stromal genes and a third showed mixed information.^40^ In relation with the difference in genetic predisposition involving T-cell genes and the autoantibody status, the first group may be speculated to reflect pathology associated with the presence of ACPA although this was not investigated in this paper. Studies combining gene expression and modern imaging could also reveal association between molecular signatures (for example, involving neo-vascularization genes) with signals in power Doppler ultrasound examination, which is predictive of progression towards early RA.^47, 48^

### Diagnostic, prognostic and preclinical signature in RA

#### Diagnostic

Given the destructive nature of RA, early diagnosis and initiation of treatment is highly important.^49, 50, 51^ The recently revised diagnostic criteria for RA (European League Against Rheumatism) 2010^52^ improved early diagnosis by including ACPA in the criteria but demonstrated even more early disease heterogeneity (only 50% of patients are positive), stressing the need for other diagnostic biomarkers for ACPA-negative disease.^53^

Using a differential display reverse transcriptase–PCR approach, genes were identified whose expression was different in early inflammatory arthritis biopsies with a diagnosis of RA as opposed to reactive arthritis.^54^ This work primarily highlighted genes that were differentially expressed in T-cell synapse (LCK (lymphocyte-specific protein tyrosine kinase), nil2a (Zinc Finger E-box Binding Homeobox 1), T-plastin (plastin isoform T)), notably in relation with anergy, which is a feature of RA (reviewed in Gatzka and Walsh^55^). These also included apoptosis-related genes (caspase), calcium signalling (calmodulin, reticulocalmin, calumenin), transcription factors, signal transduction (ADP ribosylation factors) and differentiation (Jagged/Notch), some of which were also expressed in patient's blood cells. Several genes identified in this work (calmodulin, spermine, ADP ribosylation factors, tropomyosin, eukaryotic translation initiation factors) were later confirmed using micro-array technology in synovial tissues^56^ or human blood cells.^57^ The Jagged/Notch differentiation pathway was taken forward and identified clear differences of expression in lymphocytes from RA patients compared with healthy controls and also suggested a role for this pathway in regulatory T-cell activity.^58^

Pratt *et al.* performed gene expression profiling in separated CD4+ T cells from early inflammatory arthritis patients who later went on to develop RA.^59^ A 12-gene signature distinguishing RA from non-RA patients was derived and validated by quantitative PCR (qPCR) in a second set of patients. Five out of the 12 genes belonged to the IL-6-mediated STAT3 pathway. Interestingly, this signature was valid in both ACPA-positive and -negative inflammatory arthritis patients progressing towards RA, showing an 85% sensitivity and 75% specificity for progression. Such 12-gene signature, if replicated, may serve as alternative diagnostic biomarker, particularly in seronegative patients.

#### Preclinical RA

Recognition of the preclinical phase of RA has initiated a whole new field of research aimed at the discovery of predictive biomarkers for the development of arthritis.^60^ Systemic autoimmunity was shown to precede synovitis development.^61^ Indeed, ACPA can be present for years before disease onset.^62, 63^ In contrast, synovial abnormalities (that is, increased synovial cellularity) do not occur until symptom onset,^61^ suggesting that other unknown triggers/events/location are driving the inflammatory immune response leading to RA. Alternatively, counter-regulatory mechanisms suppressing disease development despite the presence of autoimmunity may exist. Animal models have suggested that the onset of arthritic disease is preceded by phenotypic changes in draining lymph nodes (LN).^64, 65, 66^ Flow cytometry data in LN biopsies from ACPA positive at risk individuals, early arthritis patients and healthy controls suggested increased T-cell activation in early arthritis but not in ACPA-positive individuals.^67, 68^ These data support the rational for further extensive molecular analysis of LN during different phases of (preclinical) arthritis.

Gene expression profiling of peripheral blood cells in arthralgia patients (with confirmed absence of synovitis) and ACPA positivity highlighted a gene signature, including interferon (IFN)-mediated immunity and cytokine/chemokine activity that were specifically observed in at-risk individuals who then went on to develop arthritis.^69^ A second signature, including increased expression of B-cell-specific genes, appeared to be associated with protection from arthritis development. The increased IFN activity in the preclinical phase of RA was confirmed in pre-onset RA patients (samples from the Medical Biobank of Northern Sweden).^70^ The combined analysis of such IFN and B-cell signature in an independent validation cohort of seropositive arthralgia patients confirmed a significant high risk for arthritis development in IFN-high/B-cell-low profile (80%, odds ratio 6.22) and a low risk for IFN-low/B-cell-high profile (26%, odds ratio 0.16). To demonstrate clinical utility, a receiver operator characteristic (ROC) curve was constructed for ACPA+/RF+ (rheumatoid factor positive) alone and in combination with both signatures. The area under the curve increased substantially when including the IFN and B-cell signatures and the sensitivity to diagnose pre-clinical RA increased (from 16% to 52%) with a cutoff of 94% specificity.^70, 71^

#### Prognostic

Another important need in RA biomarker research relates to prognostic factors associated with disease progression and development of (new) erosions. Reynold *et al.*^72^ performed an extensive gene expression profiling study in peripheral blood cells patients with early RA (disease duration <2 years, 75% ACPA+). Using a 48-K Illumina chip (Illumina, San Diego, CA, USA), gene expression signatures could be associated with severe disease (>10 erosions) at baseline and after 36 months of follow–up; however, no clear expression profile could be associated with disease progression and the development of new erosions. Interestingly, pathway-level analysis suggests a relationship between CTLA-4 (cytotoxic T-lymphocyte antigen 4) T-cell signalling and erosive disease, consistent with a T-cell-mediated pathogenesis early in the disease. In perspective of the genetic association between several T-cell processes and ACPA+/shared epitopes positive disease,^73^ the increase in radiographic progression in these patients is most likely reflected by this gene signature.^35, 36^

### Gene expression signatures as predictors of treatment response

The expanding range of various biological therapies that is available for RA patients after failure to DMARDs like methotrexate (MTX) has led to the detection of a significant inter-patient heterogeneity in efficacy as well as in the appearance of important adverse effects of these treatments. The pro-inflammatory cytokine tumor necrosis factor α (TNF-α) has been identified as a pivotal factor in driving inflammation in RA; however, the spectrum of clinical responses to TNF blockade suggest that TNF has an important role in the early phases of disease development (with high response rate) but much less in late RA with the development of alternative TNF-independent pathways.

The first approved target for biological therapies in RA was TNF-α. Three antibodies (infliximab, adalimumab and golimumab), one Fab' antibody fragment (certolizumab) and one soluble receptor with affinity for TNF-α and lymphotoxin-α (etanercept) were developed over the years. Other biologicals targeted the cytokine IL-1 (anakinra, soluble IL-1 receptor antagonist), the cytokine receptor for IL-6 (tocilizumab, antibody), the B7 family of costimulatory molecules (abatacept, FcIgG1–CTLA4 fusion protein) and the pan B-cell marker CD20 (rituximab, antibody). Biologicals are prescribed on a trial-and-error basis and approximately 30–40% of patients fail to respond to these therapies.^74, 75^ Considering the high cost of biologicals and the possibility of severe side effects, the identification of responders and non-responders to a specific biological would be a major improvement in the management of RA patients. With the goal of optimizing prescription of biologicals for personalized approaches, several groups have studied gene expression profiling of blood cells or synovial biopsies to determine molecular signatures that would predict response to a biological therapy (Table 2).

#### Methotrexate

The mechanism of action of MTX (a folate antagonist) is still largely unknown, while it is the most commonly used drug for the treatment of RA. Its mechanism of action with respect to the folate metabolism remains elusive in RA. Blits *et al.*^76^ investigated the cellular pharmacological impact of MTX on peripheral blood cells using the samples obtained from MTX-naive and MTX-treated RA patients as well as from healthy controls. The data revealed that multiple folate metabolism-related genes were consistently and significantly altered between the three groups. Concurrent with an immune activation gene signature, a significant upregulation of folate metabolizing enzymes (γ-glutamyl hydrolase, dihydrofolate reductase), and MTX/folate efflux transporters (ABCC2 and ABCC5) was observed in the MTX-naive group compared with healthy controls. Strikingly, MTX treatment normalized such differential gene expression levels to those observed in healthy controls. Hence, these results suggest that under inflammatory conditions basal folate metabolism in blood cells of RA patients is markedly upregulated, whereas MTX treatment restores normal folate metabolism levels. This provides insight into the mechanism of MTX action, paving the way for the development of novel folate metabolism-targeted therapies, although more work in this area will be required to determine if a MTX predictive response signature can be established.

#### Infliximab

Infliximab was the first biological approved for RA, and as such, it has been mostly studied for the identification of gene expression signatures able to predict its response. In 2006, Lequerré *et al.*^77^ analyzed gene expression profiles in peripheral blood mononuclear cells (PBMCs) of RA patients before and after infliximab treatment using an arbitrary collection of 12 000 probes covering the PBMC transcriptome. They obtained a list of 41 transcripts of which 20 were confirmed by qPCR as predictors of responsiveness with performance of 90% sensitivity and 70% specificity. They next determined that eight was the minimal number of transcripts able to predict responsiveness with 80% sensitivity and 100% specificity. This publication supported the possibility that a test based on gene expression biomarkers can be designed to optimize prescription of infliximab in RA patients; however, independent validation of these results still remains to be established. The same year, another study was published looking at gene expression profiles in synovial biopsies. Lindberg *et al.*^78^ analyzed RA patients before and after 2 months infliximab therapy using an in-house microarray. Two hundred and seventy-nine differentially expressed genes were detected between responders and non-responders. At the synovium level, the MMP-3 was significantly upregulated in responders. Two years later, van der Pouw Kraan *et al.*^79^ analyzed gene expression profiles in RA synovial biopsies using a genome-wide microarray. Their main conclusion was that patients with a high tissue inflammation signature were more likely to benefit from infliximab treatment. In another article by Lindberg *et al.*,^80^ gene expression profiles in RA synovial biopsies were analyzed using an in-house microarray. The presence of lymphocyte aggregates was observed and correlated with response to infliximab confirming previous data obtained using histology.^81^ However, these results also questioned microarray analysis of whole synovial biopsies as an appropriate tool to predict the response to infliximab treatment, by stressing out that the presence of lymphocyte aggregates may represent an important confounding factor in gene expression analysis.

Whole blood appears to be better suited to study gene expression signatures and has then been extensively used to develop predictive biomarkers to infliximab response. In 2010, van Baarsen *et al.*^82^ performed a qualitative and quantitative pharmacological study of the response to infliximab in whole blood using a genome-wide microarray. In all treated patients, they observed a neutralization of bioactive TNF irrespective of the clinical response with several biological pathways being downregulated (inflammation, angiogenesis, B- and T-cell activation). These results implied a common effect of drug on TNF-driven/-related pathways independently of response; however; no predictive signature could be identified, hence suggesting parallel TNF-dependent and TNF-independent disease mechanisms. Sekiguchi *et al.*^83^ analyzed gene expression profiles in whole blood using a microarray of 747 genes selected from public database of SAGE (serial analysis of gene expression) for activated T cells, dendritic cells, monocytes and macrophages. Eighteen genes differentially expressed between responders and non-responders were identified over the course of treatment, but these genes did not predict response before treatment. They, however, reported a clear difference in the kinetics of IFN-related genes during infliximab treatment between responders and non-responders. A further study measuring 15 gene of a pre-determined set of IFN-response genes using real-time PCR in an independent group of patients before and post treatment and showed an increase in type-I IFN response gene expression 1 month after treatment in patients who had a poor clinical response to treatment.^84^ However, again, no association between response and baseline IFN response gene activity could be identified. This signature was therefore not appropriate for evaluation of treatment outcome before infliximab initiation but still may have value very early in the course of treatment.

The first blood-based gene expression signature predicting treatment response was published by Tanino *et al.*^85^ A whole human genome microarray was performed on whole blood at baseline. A list of 10 genes was identified. This 10-biomarker set was then validated with an accuracy of 66.7%. About the same time, Julià *et al.*^86^ analyzed gene expression of whole blood using a whole human genome microarray. A robust eight-gene predictor signature was identified and applied to an independent validation set of patients. The predictive performance of this signature was 94.4% sensitivity and 85.7% specificity. However, also here independent validation of these results still remains to be established.

#### Other anti-TNF agents

Stuhlmüller *et al.*^87^ analyzed gene expression profiles in monocytes of RA patients and healthy subjects before and during Adalimumab using a commercial microarray. CD11c was identified as a response to treatment biomarker and was validated by qPCR in a second cohort with 100% sensitivity and 91.7% specificity. In another study, Badot *et al.*^88^ analyzed gene expression profiles before and after adalimumab in synovial biopsies using a commercial microarray. Four hundred and thirty-nine genes were differentially regulated between poor responders as compared with the moderate and good responders and were clustered into two specific pathways: cell division and regulation of immune responses (in particular, cytokines, chemokines and their receptors). A validation study was performed by immunostaining on synovial samples and confirmed the differential baseline expression of five genes, notably CD11c.

Etanercept was evaluated for molecular discrimination between responders and non-responders. Koczan *et al.*^89^ analyzed gene expression profiles in PBMCs using a commercial microarray. Forty-two genes were differentially expressed. Twenty selected genes were studied by qPCR, and eight were able to discriminate between responders and non-responders. However, the authors concluded that even if gene expression profiling was able to identify genes that were associated with therapeutic outcomes, a gene expression signature at baseline was not reliable in predicting the clinical outcome.

#### Rituximab

Despite the effective depletion of circulating B cells in nearly all treated patients, 40–50% of patients do not respond to rituximab treatment. Julià *et al.* analyzed gene expression profiles in whole blood, CD4+ T cells, and B cells using a commercial microarray and qPCR validation. An association of TRAF1 (TNF receptor-associated factor 1) and ARG1 (Arginase 1) expression in whole blood and an association with TLR4 (Toll-like receptor 4) expression in CD4^+^T cells were observed.^90^ They also reported that the serological status of the RA patient has no predictive value for rituximab outcome. Until now, a study wherein the use of these markers is validated is not reported.

Recently, Raterman *et al.*^91^ analyzed the gene expression profiles in whole blood using a commercial microarray. They identified a type I IFN signature constituting a predicting biomarker of response. Good responders had a low or absent IFN response activity at baseline, whereas non-responders display an activated type I IFN system before the start of treatment. Such an association was in line with previous findings, demonstrating that patients with a low IFN signature had a significantly greater reduction of disease activity and more often achieved a significant response.^92^ Clinical utility as predictor of non-response to rituximab was demonstrated in a validation study using ROC curve analysis, based on an optimal set of 3–5 IFN type I genes according to the differential disease activity score.^91^ These results suggest that RA patients with an IFN^high^ signature represent a different pathogenic subset of patients. Ultimately, these results may provide a biomarker that can be implemented in clinical practice to prescribe the most effective therapy for a particular patient.

Vosslamber *et al.*^93^ studied the pharmacological effects of rituximab in RA and observed an increase in IFN response activity after treatment in responders, whereas the IFN signature remained stable in non-responders. The IFN signature score returned to baseline values at 6 months after the start of treatment in responders. Thus a pharmacological increase in IFN response activity during rituximab treatment may be necessary for a favourable response and may provide an insight in the biological mechanism underlying such response. These findings notably provide a basis for further study on the role of the IFN signature as a biomarker for effective dosing and timing of treatment, towards patient-tailored treatment and prevention of relapse.

#### Anakinra

Finally, a gene expression signature for IL-1RA (anakinra) treatment outcome was proposed. Bansard *et al.*^94^ analyzed gene expression profiles in PBMCs using an in-house microarray. Fifty-two transcripts linked to a gene network focused on IL-1β were identified of which 20 transcripts were selected for qPCR validation. Performance of this 20 transcripts signature for discrimination between responders and non-responders showed 80% sensitivity and 87.5% specificity. Seven out of the 20 transcripts were required for an acceptable prediction of response. Two publications sharing exactly the same technical features (same array and batch of PCR-amplified probes for validation)^77, 94^ allowed for a direct comparison between anakinra- and infliximab-response signature. No overlap between anakinra- and infliximab-selected transcripts was observed, indicating that the selected signature reflects drug specificity rather than pathophysiological mechanisms. However, studies on larger cohorts are needed to validate this hypothesis.

#### Tocilizumab

Tocilizumab, a humanized monoclonal antibody against the interleukin 6 receptor, is the most recent addition to the panel of antibodies used for the treatment of moderate-to-severe RA. Many clinical studies have been performed so far, and clinical responses were clearly established.^95, 96, 97^ So far, one publication reports gene expression for tocilizumab. PBMC gene expression was assessed before and 4 weeks after treatment in a small group of patients.^98^ Four genes (*CCDC32*, *DHFR*, *EPHA4* and *TRAV8-3*) were associated with response, and another 59 showed changes with treatment. The four predictive genes for response were not included in any of the published response signatures for antibody-mediated TNF blockade.

#### Conclusion and future need for validation

Not surprisingly, maybe gene expression signatures for a given clinical end point published to date do not always overlap and have not always been re-validated. The only exceptions were the signatures for progression from preclinical phase of RA^69, 70^ and the prediction of response to rituximab.^91, 92^ The often observed lack of reproducibility can be explained by the heterogeneity in technical protocols and microarray platforms used (notably when comparing in-house array to commercial ones), data analysis methods and, most importantly, the choice of clinical assessment of response to treatment (Table 3). Beside technical issues, the heterogeneity of the RA disease itself may be largely responsible for lack of replication in data generated by different groups also taking into consideration the small number of patients included in these studies.

As highlighted in the previous section, genetic risk associated with RA cover over 50–60 genes.^28, 29, 30, 31^ Work related to pathogenicity has clearly shown that different pathways can be involved at different stage of the disease evolution. Altogether, reproducibility of data could, maybe, only be expected if patients were analyzed taking into account disease duration, current and past treatment, outcome measures as well as ethnical origin, all of which suggesting the need for large numbers of patients. Finally, lack of reproducibility itself is not uncommon in microarray studies.^99^ A recent publication of interest found an association between a polymorphism in the CD84 gene with response to Etanercept. Utilizing publicly available gene expression data from PBMCs, they found an expression quantitative trait loci between the lead genome-wide association study single-nucleotide polymorphism and CD84 transcripts. Independent analysis of CD84 expression in PBMCs from microarray data demonstrated a non-significant association with response to Etanercept.^100^ Approaches that integrate genotypic and gene expression data may therefore aid in the interpretation of genotype–phenotype relationships.

An external validation study^101^ of existing gene expression signatures for anti-TNF treatments in RA was performed using a genome-wide expression profiling to validate eight previously reported signatures predicting anti-TNF therapy outcome (Lequerré *et al.*^77^—20 and 8 genes, Stuhlmüller *et al.^87^*—82, 11 and 3 genes, Julia *et al.*^86^—8 genes, Tanino *et al.*^85^—8 genes and Sekiguchi *et al.*^83^—18 genes) on whole blood of 42 RA patients before treatment with infliximab or adalimumab. One hundred and thirteen genes differently expressed between responders and non-responders at baseline were identified. Although the signature of Lequerré *et al.*—20 genes was validated with 71% sensitivity and 61% specificity, the robustness of the validation can be debated given that only one algorithm was used to reach that conclusion. Nevertheless, this work clearly demonstrate the need for standardization of any signature across different groups and suggest that proofs of concept exist but that collaboration to perform meta-analysis bridging between different technical platforms will be needed to bring this data into usable biomarker signatures with respect to individual drug (and/or target) specificity.

One major drawback of these studies remains the small number of patients analyzed and the absence of internal validation for most of them. On the other hand, it may be possible to develop a global signature able to predict response to biologicals that share the same target. Bienkowska *et al.*^102^ analyzed gene expression profiles in whole blood before etanercept, infliximab or adalimumab using a commercial microarray. An eight-gene transcript signature that predict response to three TNF-α blockers was identified with 89% accuracy (using the Convergent Random Forest approach, for selection of non-redundant molecular features predictive of treatment response, in order to identify the minimum number of features with a maximal predictive power). This observation is in line with data from Meugnier *et al.*^103^ comparing the effect of etanercept or adalumimab on leukocyte gene expression in RA patients who successfully responded to treatment.^102, 103^

### miRNA signatures in RA

MicroRNAs (miRNAs) are a recent addition to the gene regulatory mechanisms,^104, 105^ and researchers are now exploring the possibility of measuring miRNA in serum samples as well as cells. miRNA represent a class of non-coding RNA molecules having pivotal roles in cellular and developmental processes, predicted to affect up to 1/3 of all human protein-encoding genes. miRNAs are potent negative modulators of gene expression involved in several cellular processes.^106^ Compared with other gene regulatory mechanisms such as epigenetic and transcription factors, miRNA-mediated effects allow for the fine tuning of gene expression. Downregulation of gene expression occurs by binding to complementary sites in the 3′ untranslated region of target mRNAs leading to the degradation of mRNA.^107^ miRNAs are considered to exert fine modulation rather than switch on/off mechanisms in the regulation of genes. In addition to regulating biological processes such as development, proliferation, apoptosis, hematopoiesis, angiogenesis, metabolism, anti-viral and immune responses, miRNA research gained widespread attention with the recognition of aberrant expression and/or function in cancer, neurological, cardiovascular, metabolic, neurodegenerative, infectious, chronic inflammatory and autoimmune diseases.^108, 109, 110, 111, 112, 113^

In terms of biomarker value, increasing numbers of reports implicate an aberrant expression of certain miRNAs (miR-21, -17, -92, -15, -16, -18a, -103, -107 -141, -193) in response to chemotherapy in different malignancies while, in parallel, they can be used for the classification of patients in different subgroups.^114, 115, 116, 117^ A large study on miRNA expression in SLE determined several patterns of differentially expressed miRNAs involved in various biochemical pathways leading to different subtypes of diseases.^118^ miRNA seem to be important players in RA as well.^107, 119, 120, 121^ They were shown to modulate the inflammatory process in joints (miR-155, miR-146).^122, 123^ Further, differential miRNA expression was found in RA compared with OA, in relation to inflammation, and the target genes involved were related to cartilage and bone damage.^123^ Comparing miRNA expression in RA and OA synovial fibroblasts revealed subsets of miRNAs that could potentially be used as clinical markers.^124^ OA fibroblast-like synoviocytes expressed a considerably lower level of miR-124a than RA, with effect on proliferation and chemokine production.^125, 126^ In the peripheral blood of RA patients, miR-146, miR-155 and miR-16 were upregulated, in relation to active disease,^123, 127^ suggesting a potential as markers of disease activity.^112^ miR-346 negatively regulated the IL-18 response of RA fibroblast,^128^ whereas miR-146a was found to be associated with IL-17 expression in both PBMC and synovium of RA patients.^129^ In turn, TNF and IL-1 were shown to regulate the expression of miR-146a in RA fibroblast-like synoviocytes.^130^ Of note, a methylation-dependent regulation of miR-203 expression in RA synovial fibroblasts has been demonstrated.^131^

Recently, miRNA biomarkers present in plasma with diagnostic potential were also reported^121^ (miR-24, miR-30a-5p and miR-125a-5p), compared with OA and SLE.

To our knowledge, there has been no study yet investigating whether altered expression of any miRNAs is involved in RA patients' response to therapy; however, these will most likely become available in the near future. Having defined such signature will allow either these miRNAs or their corresponding gene targets to be examined for any potential correlation with response to anti-TNF or other biologicals treatment in RA. These results will clearly uphold the basic studies associating gene polymorphisms and gene expression with the response to treatment, towards an improved personalized medicine approach in RA.

### Ethical issues associated with development of gene expression signature in RA: towards personalized medicine

Gene expression signatures represent molecular fingerprints that underlie human disease, with the potential to suggest an outcome with the highest probability of achievement.^132, 133^ The use of molecular tools raises several ethical and legal concerns. Not all clinical studies involve the same risks; the number of ethical and legal issues in biomedical research, therefore, depends on the nature of the test/data involved. Long-term implications for health were associated with genetic data;^134^ however, data derived from transcriptome analysis, as well as proteomics and metabolomics, carry less ethically charged information than measures of the genome. Hence, the testing of gene expression appears much less problematic in terms of ethics due to the fact that mRNA gene expression analysis is functionally close to any other biochemical biomarker or dynamic phenotype and is not a permanently affixed label to its carrier.^135^ Therefore, identifiable data are less sensitive.

The initial hope that the experiment will benefit the patient need to be carefully addressed, especially when very sick patients are eager to accept the promise of an unproven test. How we should use gene expression profiling to guide decisions on preventative and therapeutic intervention without over treating (thus probably leading to adverse effects) remains difficult to regulate, and experience will most likely direct future guidelines, especially considering the potential conflict of interest between commercial companies offering preclinical testing and those offering interventions. Once the initial research phase has validated the signature, it becomes essential to determine ‘who' should participate in the final decision to treat the patient (in the case of a therapy outcome signature). Hence, there must be reasonable scientific evidence that the patient will benefit from the test being performed, as well as acceptable risks that one will not. This involves understanding and explaining the strengths and limitations of the signature to the patients that is critical to the formulation of information leaflet for safe and effective use of the test results.^136^ Result disclosure to patients participating to that research phase must also be resolved from the initial consent. An important consideration is the problem of constructing an accurate prediction rule, and overly optimistic estimations of prediction performance must be avoided.^136^ Patients need to be aware of real expectations. Indeed, the concept of significance of a test is unknown to the people who are tested, and they often literarily do not understand what they are getting into, hence it may carry a psychosocial impact the person is not prepared for.

Considering that the costs of obtaining data for gene expression microarray tend to be very expensive and could be reduced by merging existing data sets, data sharing, therefore, appears essential to allow the development and validation of gene expression signature. Similarly to informed consent, data-sharing approaches must also be established initially to favour collaborative work. In terms of developing a predictive tool, result-sharing approaches will have to be used.^132^

### The future of gene expression tests in RA

It is clear from the above review that gene expression profiling offers great potential for understanding RA biology as well as for patient management and personalization of treatment decisions. It usually takes many years for new biomarkers or sets of biomarkers to reach clinics, starting from basic research biomarker discovery followed by validation, clinical utility evaluation, manufacturing development and final approval by regulatory authorities. This is further complicated by the difference in legislation between European Union, United States and other part of the world, although some recent approaches are aiming to accelerate translation of biomarker from bench to clinic.^135^ In the case of novel gene expression-based test, there is a further need for clear and specific regulations. The Minimum Information About a Microarray Experiment (MIAME) guidelines were developed by the Microarray Gene Expression Data Society and published in 2001 with such a goal in mind.^137^ The MIAME guidelines lay out the minimum standards needed to ensure that an experiment using microarrays can be properly and independently interpreted.^138^ Several clinical application tests using gene expression profiling assays are already available on the market. It seems, however, that these commercialized multi-gene prognostic and predictive tests are so far limited to the field of breast cancer and other cancer diagnostics. Some are FDA approved and already support clinicians in prediction of tumour reoccurrence and making decisions in selection of treatment (MammaPrint, Oncotype DX),^139, 140, 141^ but for others the evidence supporting clinical utility is still limited (THEROS Breast Cancer Index, Aviara MGI, Mammostrat, BreastOncPx and PAM50).^21, 142, 143, 144, 145, 146, 147, 148, 149^ Hence, none of these are currently recommended for the evaluation of breast cancer recurrence risk by national guidelines (that is, National Comprehensive Cancer Network or American Society of Clinical Oncology), and these tests remain considered as investigational. Recently, new tests emerged for acute heart allograft rejection (Allomap XDx, FDA approved)^24, 150^ for diagnosis of obstructive CAD (obstructive coronary artery disease) in patients with chest discomfort (Corus CAD)^151, 152^ and for the detection of Alzheimer's disease.^153, 154^ As all novel biomarkers, it will take time for these to be adopted in daily clinical practice despite recommendation and guideline being published by professional association.^155^

Thus, there is precedent that could be applied to RA and other autoimmune diseases. The Rheumatology community is therefore facing a challenge to pool together the necessary resources and to use the available information already collected to reach appropriate conclusions and proceed with further validation, which is necessary to reach the next level. Considering the impact of RA on our modern society and the large amount of data already available, regulatory authorities such as the European Commission (FP7 and 2020 Horizon programs) as well as industry (Innovative Medicine Initiative) have developed grant schemes to overcome this challenge. Clear guidelines and standardization for such new technology have also been developed (MIAME guidelines).^138^ The successful translation of microarray data into clinical use in cancer provides a strong rational for a similar approach to improve the care of patients in rheumatology.

## Figures and Tables

**Table 1:** Gene expression studies investigating the pathogenesis of RA

*Number of samples*	*Technology*	*Analysis methodology*	*Comparison*	*Important findings*	*Reference*
*Synovium*					
21 RA 9 OA	In-house cDNA 18000 spots representing genes of relevance in immunology	Hierarchical clustering Tree view Significance analysis of microarrays (SAM)	RA vs OA Within RA	Multiple pathways of tissue destruction and repair	van der Pouw Kraan *et al.*^40^
15 RA	In-house cDNA 11 500 genes	Clustering Tree view SAM	Within RA	160 genes distinguishing two RA subsets	van der Pouw Kraan *et al.*^156^
5 RA 10 OA	In-house cDNA 5760 genes	Clustering Tree View	RA vs OA	Genes differentially express between RA and OA	Devauchelle *et al.*^56^
12 early RA 4 late RA	In-house cDNA 23 040 genes	Clustering Tree View	Early vs longstanding RA	Early RA divided into two groups based on differences in genes critical for proliferation and inflammation	Tsubaki *et al.*^44^
13 RA	In-house cDNA 29 717 genes	Expression Analysis Systematic Explorer Hierarchical structure of gene ontology	Intra- and inter-individual patients	Gene expression differences between patients are greater than between biopsies obtained from the same joint	Lindberg *et al.*^157^
12 RA	In-house cDNA 11 500 and 18 000) and qPCR	SAM hierarchical Clustering Tree View gene set enrichment analysis using pathways	Within RA	IL-7 signaling pathway lymphoid neogenesis	Timmer *et al.*^158^
12 RA 10 OA 9 HC	Affymetrix (Santa Clara, CA, USA) 54 000 probes	MAS 5.0 software	Inter-individual variances in RA, OA and HC	Disease-relevant pathways of RA pathogenesis in different individuals depend less on common alterations of expression of specific key genes than on individual variation	Huber *et al.*^159^
66 RA 51 OA 72 HC	Affymetrix 15 000 clones	SAM, hierarchical clustering	Within RA RA vs Oa RA vs ND	Molecular signatures between: RA and OA RA and HC OA and HC	Ungethuem *et al.*^160^
17 RA	cDNA microarrays ∼20 000 unique genes	Cluster analysis, TreeView, SAM, pathway analysis PANTHER	Within RA	Gene expression differentiated RA synovial tissue into high and low inflammatory subgroups. Histological tissue subclassification matched the subclassification based on gene expression analysis. This subclassification was not reflected in peripheral blood samples	van Baarsen *et al.*^46^
					
*Fibroblast-like synovial cells and cell cultures*					
5 RA 5 HC	Atlas cDNA array 588		RA vs HC	Tumour-like growth pathways	Watanabe *et al.*^161^
19 RA	18 000 genes	SAM hierarchical clustering	Within RA	Heterogeneity between patients is reflected in FLS cultures (passage 4)	Kasperkovitz *et al.*^162^
17 RA 20 OA 6 HC	Affymetrix	ArrayAssist	RA vs OA and HC	Expression heterogeneity between patients with the same disease Home box-specific patterns for RA Six gene signature specific for OA FLS signature is related to clinical characteristics with respect to diagnostic, prognostic and response to treatment	Galligan *et al.*^43^
					
*Whole blood*					
35 RA 15 HC	18 000	SAMhierarchical Clustering Tree View gene ontology analysis	Within RA and vs HC	Increased type I IFN signature in a subpopulation of patients	Van der Pouw Kraan *et al.*^163, 164^
109 RF+ve and/or ACPA+ve arthralgia patients 25 RA 6 HC	cDNA microarrays ∼20 000 unique genes. Taqman low-density arrays	SAMCluster analysis, PAM PANTHER	Within arthralgia patients and vs HC and RA	Identification of gene expression profiles (IFN-mediated immunity and B-cell activity genes) predictive for the progression to arthritis in autoantibody-positive individuals at risk for developing RA	van Baarsen *et al.*^69^
115 sero+ve arthralgia 25 sero+ve no symptoms 45 HC	Fluidigm (Fluidigm Corporation, South San Francisco, CA, USA)		Within arthralgia population converters and non-converters to arthritis	Seven IFN gene signature as predictors of progression to RA	Lübbers *et al.*^70^
					
*Peripheral blood mononuclear cells*					
14 RA (8 RF+ve, 6 RF−ve) 7 HC	10 000		Within RA (RF+ve vs RF−ve) RA vs HC	No genes differentially expressed between RF+ and RF− Increased expression of immune-inflammatory response genes, phagocytic functions	Bovin *et al.*^165^
19 RA 14 SLE 11 asthma 9 post vaccine	4300	Cluster analysis	RA vs other diseases Within RA	Early stage of RA is associated with a distinct gene expression profile in PBMCs subset of patients with SLE shared the ERA signature	Olsen *et al.*^166^
29 RA 21 HC	12 626	Hierarchical clustering	RA vs HC	Monocyte-associated gene signature	Batliwalla *et al.*^167^
18 RA 15 HC	Illumina 48 701	Supervised hierarchical clustering Tree view Gene Ontology analysis	RA vs HC	Increased biological mechanism: immunity and defense. No significant downregulated ontology groups Biomarkers for diagnostic interventions Biomarkers for therapeutic interventions	Teixeira *et al.*^168^
23 RA	In-house 4500 cDNA sequences	SAM	Within RA	29 gene signature for SE+ve RA 91 gene signature for active RA (DAS28>5.0) 101 gene signature for CCP+ve RA	Junta *et al.*^169^
49 RA 50 SpA 17 OA HC	TaqMan custom-made array	MedCalc software package	RA vs SpA, OA, HC	Bone metabolism signature in blood form RA/OA/SpA	Grcevic *et al.*^170^
17 early RA 9 established RA	In-house cDNA 4133 cDNA	SAM, hierarchical clustering	Early vs established RA	19 gene signature of disease severity in patients with early and established RA	Liu *et al.*^171^
96 RA	Illumina 25 000 cDNA	Ingenuity Pathways Analysis	RA baseline vs 36 months	Significantly correlated with total erosions at baseline but not with change in erosion over time No evidence of a signal differentiating disease progression	Reynolds *et al.*^72^
					
*B-cells/ lympho-blastoid cell lines*					
8 RA + 8 HC	In-house 21 329	Pathway Analysis (Pathway Assist software)	RA vs HC	Dysregulated B-cell biology Pathogenic humoral immune response	Szodoray *et al.*^172^
11 pairs of RA-discordant MZ twins	Microarrays 20 000 gene	Significance analysis of microarrays Tree View Gene ontology	RA twin vs the healthy twin	Many discordant genes (upregulated and downregulated)	Hass *et al.*^173^
					
*Bone marrow-derived mononuclear cells*					
9 RA 10 OA	Affymetrix	Expression Analysis Systematic Explorer (EASE) Ingenuity Pathway Analysis	RA vs OA	Abnormal regulatory networks in the immune response Indication that the BM is pathologically involved in RA	Lee *et al.*^174^

Abbreviations: ACPA, anti-citrullinated protein antibody; BM, bone marrow; FLS, fibroblast-like synovial cells; HC, healthy controls; MZ, monozygotic; IFN, interferon; OA, osteoarthritis; PBMC, peripheral blood mononuclear cell; RA, rheumatoid arthritis; RF, rheumatoid factor; SE, shared epitopes; SLE, systemic lupus erythematosus; SpA, spondylo arthropaty.

**Table 2:** Gene expression signatures as a tool for treatment outcome prediction

*Drug*	*Tissue*	*Number of samples*	*Technology*	*Software*	*Comparison*	*Results*	*Reference*
MTX	CD4+ T-cells	31 early RA	Illumina	GeneSpring XI (Agilent Technologies, Santa Clara, CA, USA)	Responders vs non-responders	133 CD4+ T-cell transcripts differentially expressed	EWRR 2013 Abstract Pratt *et al.*^175^
	Whole blood	52 RA	Affymetrix	Hierarchical clustering	Responders and non-responders	16 gene signature shows clear discrimination between responders and non-responders to fMTX treatment	EWRR 2013 Abstract Mans *et al.*^176^
	Synovial tissue fibroblast cells (FLS)	17 RA; 20 OA; 6 HC	Affymetrix 47 000	Hierarchical clustering, gene ontology classification	RA vs OA, within RA	Different profiles in RA and OA FLS. Eleven genes elevated in RA on MTX 23 genes upregulated in RA on prednisone therapy. Prednisone and MTX treatment affected gene signatures	Galligan *et al.*^43^
Leflunomide	PBMC	10 patients with early RA	DualChip 282 genes	Hierarchical cluster, statistical environment “R”	Before vs 12 weeks after treatment	Treatment of early RA Downregulation of many genes	Soldana *et al.*^177^
IFX	Synovium	10 RA	In-house array 30 000 cDNA spots	Hierarchical cluster, statistical environment ‘R'	Before vs after 9 week of treatment	Genes specifically changed in patients who have a good response to IFX treatment	Lindberg *et al.*^78^
	Synovium	18 RA	Human cDNA microarrays 18 000	Supervized hierarchical clustering. Tree view gene ontology analysis (PANTHER database)	Responders vs non-responders to IFX	Patients with high expression of genes involved in tissue inflammation before treatment are more likely to benefit from IFX therapy	van der Pouw Kraan *et al.*^79^
	Whole blood	18 RA	Customized microarray 747 genes	Cluster analysis	Responders vs non-responders	Unique set of genes with differentially expressed in responders and non-responders to IFX	Sekiguchi *et al.*^83^
	Whole blood	44 RA	Illumina 47 000	Statistical environment ‘R'	Responders vs non-responders	Eight-gene signature predicting response to IFX	Julia *et al.*^86^
	Whole blood	Discovery set 42 RA, validation set 26 RA	Agilent 44 000	Gene ontology	IFX vs MTX responders vs non-responders	10 gene signature for response 65.4% accuracy of prediction	Tanino *et al.*^85^
	PBMC	13 RA	In-house 12 000 cDNA	Hierarchical clustering SAM	Before vs 3 months after responders vs non-responders	Predictive signature for IFX/MTX efficacy Profile correlating with treatment response	Lequerré *et al.*^77^
	PBMC	23 RA	In-house array 4500 cDNA	Significance analysis of microarrays (SAM)	IFX treated vs non-treated with IFX	28 signature exclusively expressed group treated with DMARDs+IFX	Junta *et al.*^169^
	Synovial tissue	62 RA	In-house array 17 972 unique genes	SAM), hierarchical clusters, gene ontology	responders vs non-responders	Feasibility study	Lindberg *et al.*^80^
	Whole blood	Discovery set 15 RA, validation set 18 RA	20 000 unique genes	Cluster analysis, Tree view ontology (PANTHER)	Before and 1 months after IFX treatment	Downregulation of genes in several biological pathways Inflammation Angiogenesis B- and T-cell activation Pharmacological response signature	Van Baarsen *et al.*^82^
	Whole blood	33 RA	In house: 43 000 cDNA qPCR TaqMan	Tree view Ontology (PANTHER)		Candidate 34 INF gene signature set Validated 15 IFN gene set by Taqman Final 5 gene set signature	Van Baarsen *et al.*^84^
	Peripheral blood	RA	RT-qPCR	Canonical Variates Analysis (CVA)	Responders and non-responders	30 gene set signature differentiated responders from non-responders	EWRR 2013 Abstract Szekanecz *et al.*^178^
IFX or ADA	Whole blood	42 RA	Affymetrix 17 881genes	K-mean clustering	Responders vs non-responders	Eight-gene signature: sensitivity of 71%, specificity of 61%	Toonen *et al.*^101^
ADA	Synovium	25 RA	Affymetrix 39 000 genes		Baseline vs 12 weeks of therapy.	Markers of response to TNF blockade	Badot *et al.*^88^
	Monocytes	Discovery set *n*=7, validation set *n*=77	Affymetrix	Hierarchical clustering; Gene Ontology; gene interaction analyses via Ingenuity Pathway Analysis	Responders vs non-responders	Increased expression of CD11c in responders to ADA: sensitivity 100% specificity 91.7% power 99.6%	Stuhlmuller *et al.*^87^
ETN or ADA	PBMCs	8 RA	25 341 genes	SAM	Responder	Signature to better understand the mechanisms of action of anti-TNF treatment in RA patients	Meugnier *et al.*^103^
ETN	PBMC	19	Affymetrix 18 400+ qPCR	Gene regulatory network	Before vs 72 h after	Gene pairs and triplets predictive for response at an early time point	Koczan *et al.*^89^
RTX	Whole blood, CD4 T cells, B cells	9 RA	Illumina+ TaqMan real-time PCR		Responders vs non-responders	Several genes were associated with response in all three blood cell types	Julia *et al.*^90^
	PBMCs	Discovery set 20 RA, validation set 31 RA	qPRC three gene signature		Clinical response	The type I IFN signature negatively predicts the clinical response	Thurlings *et al.*^92^
	Synovium	20RA	Affymetrix 39 000 genes	Pathway analyses (DAVID), Gene Ontology, Gene Set Enrichment Analysis	Baselines vs 12 week	RTX displays unique effects on global gene expression profiles in the synovial tissue	Gutierrez-Roelens *et al.*^179^
	Whole blood	13 RA+9 RA	Illumina+qPCR	SAM, clustering (treeview), Gene set enrichment analysis, MetaCore Pathway analysis	Before and after treatment	Significant differential expression of of IFN-type I response genes (IRGs) at 3 and 6 months of RTX treatment. Pharmacodynamic induction of IRG expression in responders at 3 months, which is absent in non-responders At 6 months, the IRG expression returns to baseline in the responders	Vosslamber *et al.*^93^
	Synovium	20 RA	Fluidigm	Clustering hierarchical clustering	Before and after treatment	Baseline synovial Gene Score correlates with early and late clinical responses Gene Score biology suggests that T cells and macrophages are important. Expression of remodelling and IF-α genes correlates with poor response	Hogan *et al.*^180^
	Whole blood	Discovery set 14 RA, validation set 26 RA	Illumina	SAM, hierarchial clustering (treeview), Ingenuity pathway analysis	Responders vs non-responders	Significant differential expression of IFN-type I response genes (IRGs) at 6 months of RTX treatment. Baseline prediction of non-response to RTX with a 3 and 8 IFN type I response gene signature	Raterman *et al.*^91^
TOC	PBMC	13 RA	Affymetrix 28 869 genes, qPCR	Canonical variates analysis. Tree view ontology (PANTHER)	Responders and non-responders	59 genes showed significant differences in response to treatment. Four genes determined responders after correction for multiple testing. Ten of the 12 genes with the most significant changes were validated by RT-qPCR	Mesko *et al.*^98^
ANA	PBMC	32 RA	cDNA array 12 000 probes	Hierarchical clustering	Responders vs non-responders	52 transcripts discriminating responders	Bansard *et al.*^94^
MTX and anti-TNF	PBMC	25 RA	4500 cDNA sequences	Statistical environment ‘R'	Responders vs non-responders	Differentiation of responders from non-responders to MTX and anti-TNF	Oliveira *et al.*^181^
MTX and anti-TNF (ETN, ADA, IFX)	Whole blood	60 RA (30 MTX 30 anti-TNF)	Affymetrix	Hierarchical clustering	MTX vs anti-TNF	Expression of 34 genes was associated with DAS28-CRP Expression of 16 genes differed significantly between the treatment groups	Parker *et al.*^182^

Abbreviations: ADA, Adalimumab; ANA, anakinra; ETN, Etanercept; HC, healthy controls; IFN, interferon; IFX, Infliximab; LEF, Leflunomide; MTX, Methotrexate; OA, osteoarthritis, PBMC, peripheral blood mononuclear cell; PN, prednisone; RA, rheumatoid arthritis; RT-qPCR, reverse transcriptase–quanititative PCR; RTX, Rituximab; TOC, Tocilizumab.

**Table 3:** Important records for successful comparison between gene expression studies

*Study design*	
	1. Studied populations (a) Demographics (age, gender, race) (b) Biological groupings between subjects (health or disease symptom duration and so on) (c) Diagnostic criteria, stratification, concomitant medication
	2. Differences in clinical management of the patients (that is, measurements in treatment response)
	3. Power analysis and confidence
	4. No validation/replication set of patients included in analysis
	
*Technical aspects*	
Sample collection and processing	5. Methods and timing of sample collection and processing (a) Blood tubes PAXgene vs Tempus vs PBMC (b) Influence of circadian rhythm (c) Effect of tissue handling (*ex vivo* cell-isolation procedures may activate some cell types)
Sample selection	6. Cell source (a) Blood vs tissue (b) Whole blood vs PBMCs vs isolated sub-populations of immune cells (c) Tissue anatomical differences (d) Tissue-heterogeneity of cell types within biopsy (synovial tissue, synoviocytes, tissue architecture complexity)
Analysis methodology	7. Overlap between sets of genes investigated in different studies
	8. Methods for the final selection of predictive genes (as an outcome of the study)
	9. Different algorithms used to select genes for investigation
Array selection and preparation	10. Technological variation (array platform): custom made (in-house made) vs commercially available arrays
Hybridization	11. Hybridization, mixing, washing, drying, QC

Abbreviations: PBMC, peripheral blood mononuclear cell; QC, quality control.
